# Database energy saving strategy using blockchain and Internet of Things

**DOI:** 10.1038/s41598-024-67265-6

**Published:** 2025-01-17

**Authors:** Dexian Yang, Jiong Yu, Zhenzhen He, Ping Li

**Affiliations:** Xinjiang Vocational and Technical College of Communications, Urumqi, Xinjiang 831401 China

**Keywords:** Blockchain technology, Database, Energy saving mechanism, Green environmental protection, Information technology, Physics, Energy science and technology

## Abstract

This paper aims to construct a green environmental protection system by advancing database energy-saving techniques and optimizing the energy-saving mechanism against the backdrop of blockchain integration. The protocol classification of wireless sensor networks is examined within the context of the rapid growth of information technology. The analysis draws upon the database storage and sharing model and recent research examples that connect blockchain and database technology. Additionally, the paper investigates the blockchain data structure and storage path by analyzing the spatial–temporal correlation properties of blockchain data. The findings demonstrate that increasing the number of network nodes in the database system within a reasonable range can reduce the overall execution time. Additionally, as time progresses, the query traffic of the system database continues to increase. The research has practical implications in the domains of databases and the Internet of Things.

## Introduction

Energy saving has emerged as a prominent subject in technological development and scientific research, owing to the rapid advancement of information technology and the passing of time^1–3^. The exponential increase in data generated by various infrastructures presents an urgent challenge concerning its storage and sharing. This surge is attributed to the rapid proliferation of emerging technologies such as cloud computing and the Internet of Things (IoT). Creating a database-based data life cycle model is essential to promote intelligent data sharing and openness. However, the issue of database energy consumption has attained growing importance as data storage sizes and capacities continue to expand annually. Consequently, implementing proper research techniques to optimize the database’s energy-saving mechanism can substantially enhance the database system’s data transmission efficiency^4^.

The establishment of a low-carbon society is widely accepted, driven by escalating global energy consumption and resource constraints. As information technology has advanced, computers have become integral to various aspects of social production, impacting people’s daily lives and occupations^5,6^. However, this technological progress has also brought about a corresponding increase in the significance of energy usage. Notably, carbon dioxide emissions from the computer database sector are comparable to those of the aviation manufacturing sector, and these emissions continue to rise. In response to such pressing environmental concerns, green computing has emerged to mitigate the environmental impact of computing activities.

The primary contribution of this study lies in leveraging blockchain technology to extend system resources and alleviate the storage burden of databases. A bridge is built between the high-level semantics of the blockchain and the underlying storage layer to create the energy-saving mechanism of the database. In the performance optimization of the edge server, this paper uses a wide range of device deployments to improve the system service. This approach serves as a valuable benchmark for enhancing the energy-saving capabilities of databases.

## Recent related work

### Recent studies of blockchain and database technology

Monrat et al.^7^ examined blockchain technology from the perspectives of applications, challenges, and opportunities. They highlighted its decentralized, tamper-proof, transparent, and auditable features, which enhance the security and integrity of transactions. The authors also reviewed relevant literature on blockchain technology, demonstrating its wide applicability across various industries, including risk management, healthcare, and financial and social services. Bernabe et al.^8^ investigated privacy protection solutions in blockchain by examining several scenarios in e-government and e-health. They identified scalability, security, and potential privacy issues that could pose challenges to blockchain implementation. Careful exploration of transaction linkability in blockchain was deemed crucial to address these concerns. Shahnaz et al.^9^ applied blockchain in electronic health records, emphasizing issues with blockchain data security, integrity, and management. They proposed an implementation methodology specific to the healthcare sector, providing valuable insights into the utilization of blockchain in healthcare. Blockchain research was done for Industry 4.0 by Bodkhe et al.^10^ explored cutting-edge blockchain technology options for smart applications in the context of Industry 4.0. Their findings highlighted the potential of blockchain to address scalability, resilience, and data storage issues in intelligent applications. Wang et al.^11^ investigated the potential future development of energy blockchain, emphasizing its application in the renewable energy sector. They noted that most current energy blockchain research is focused on renewable energy and that combining blockchain with the energy industry represents a novel cross-disciplinary research area with most current studies focusing on renewable energy. Qiao et al.^12^ discussed the integration of blockchain technology into digital communication and network. They focused on blockchain system architecture, consensus algorithms, smart contracts, and scalability. Additionally, the authors explored use cases of blockchain in protecting intellectual property, IoT, and digital twins. In the realm of providing triple anonymous identity trust services based on blockchain in the Industrial Internet of Things (IIoT), Li et al.^13^ proposed a fine-grained data sharing scheme for a cloud IoT system. This scheme incorporates group signatures to ensure precise access control and data integrity across multiple groups, reducing reliance on traditional trusted third parties in data public auditing. They introduced blockchain technology for distributed data public auditing, demonstrating its applicability to large-scale IoT systems. In the context of establishing trusted multidomain collaboration based on distributed blockchain in 5G and beyond mobile edge computing, Kundra^14^ designed various mobile electronic commerce (MEC) platforms using blockchain. An adaptive approach for transmission and topological privacy-preserving routers was employed. Blockchain was utilized to create multiple commitments and trust connections, facilitating cooperative traffic monitoring through membership qualification. Blockchain established multiple loyalty and trust connections through membership services and protocol methods, verifying collaborative processes. Results showed that this strategy significantly enhanced the legitimacy and effectiveness of MEC collaboration.

In recent years, blockchain technology has found widespread applications in the fields of IIoT and mobile edge computing. Umran et al.^15^ conducted a study focusing on security data issues within cement factory IIoT, leveraging blockchain technology. Their approach integrated blockchain and smart contracts to ensure data security and trustworthiness, with validation through a series of experiments. Latif et al.^16^ proposed a blockchain-based architecture designed to enhance security and trustworthiness in industrial IoT operations. Their architecture seamlessly combined blockchain and smart contracts to establish secure data storage and access control mechanisms, which were thoroughly evaluated for feasibility and performance through experimental analysis. In a comprehensive review, Yang et al.^17^ examined integrated systems involving blockchain and edge computing, delving into related research challenges and presenting potential solutions. Their review also summarized the current applications and development trends within this dynamic field. Gupta et al.^18^ delved into the realm of secure solutions that unite blockchain and edge intelligence, introducing a trustworthy and secure framework aimed at safeguarding user data and privacy. This approach skillfully integrated blockchain technology with edge intelligence principles. Resource management within cloud wireless access networks was a focal point for Rodoshi et al.^19^. Their research encompassed a comparative analysis of traditional and innovative approaches, addressing critical aspects such as resource allocation, power control, and interference management. The study proposed several enhanced methods to tackle these challenges. Suresh et al.^20^ introduced an innovative load balancing approach tailored for cloud wireless access networks. Their methodology leveraged an enhanced meta-heuristic algorithm, seamlessly integrated with mobile communication networks. The outcome was efficient resource allocation and load balancing, substantiated through a series of rigorous simulation experiments.

In summary, it is evident that blockchain technology has experienced widespread adoption in the fields of IIoT and mobile edge computing in recent years. Researchers have harnessed a diverse range of technological tools, including blockchain and smart contracts, to bolster data security, trustworthiness, and access control. They have thoughtfully tailored these solutions to cater to the unique demands of various domains. Furthermore, researchers have astutely recognized and addressed challenges and issues inherent in the application of blockchain technology within these domains, such as scalability and privacy protection. As a result, future research endeavors will likely be centered on mitigating these challenges and further refining the utilization of blockchain technology in IIoT and mobile edge computing. Moreover, there exists significant potential for interdisciplinary collaboration, where blockchain intersects with other domains such as the energy industry, digital communications, and networking. These collaborative efforts are poised to propel the advancement of blockchain technology, establishing a robust foundation for more secure, reliable, and efficient applications in the realms of IIoT and mobile edge computing.

### Research on database energy-saving mechanism

Numerous academics have made significant contributions in the realm of big data, blockchain technology, and database energy-saving mechanisms. Cai et al.^21^ focused on the data uploading process in intelligent cyber-physical systems. They proposed a heuristic approach and evaluated its efficiency using real-world data sets. The evaluation results demonstrated that the performance of the suggested algorithm aligns with the model’s ideal outcomes. Xu et al.^22^ studied the integration of big data and blockchain technology in open edge environments to establish trust by utilizing the non-repudiation and non-tampering properties of the blockchain. They developed a framework for sharing large data based on blockchain technology to support diverse applications on the server’s edge. The outcomes demonstrated that the proposed model outperforms existing approaches. Popli et al.^23^ explored the design of energy-efficient and bandwidth-restricted IoT systems, which contribute to reducing the database’s energy consumption through the IoT’s green communication. Their work is valuable for understanding how to increase energy utilization efficiency and the advancements in resource allocation technology. An energy-efficient data sensing path method in the underwater IoT was researched by Khan et al.^24^. The proposed underwater wireless sensor network (WSN) with path planning demonstrated significant energy conservation and prolonged service life through thorough quantitative evaluation. Mao et al.^25^ extensively assessed the IoT’s energy-saving communication and computer systems. They analyzed the sensor and computing services in energy-saving industrial IoT. The outcomes highlighted the substantial impact of constrained resources on the database systems’ service lives and database systems. The integration of mobile communication and edge computing technology is crucial to enhance the energy efficiency of industrial IoT.

The categorization of the WSN protocol is closely linked to the database energy-saving mechanism. Smys et al.^26^ studied the routing protocol categorization problem based on energy efficiency. Their proposed classification and comparison results emphasize the need for routing tasks to leverage various intelligent technologies to prolong the network life and ensure better coverage of perceived areas. The study examined energy-efficient protocols in WSN and demonstrated their effectiveness. In order to improve the outcomes of the urban scale building energy model, Mutani et al.^27^ investigated the urban energy model and energy efficiency scheme based on geographic information system, taking into account the actual characteristics of the built environment. Through their research, they generated a new reconstruction database to improve the accuracy and effectiveness of urban energy models.

## Study on energy-efficient database energy-saving mechanism and cluster query allocation strategy

### Analysis of spatial–temporal correlation characteristics of blockchain data

**Figure 1:**
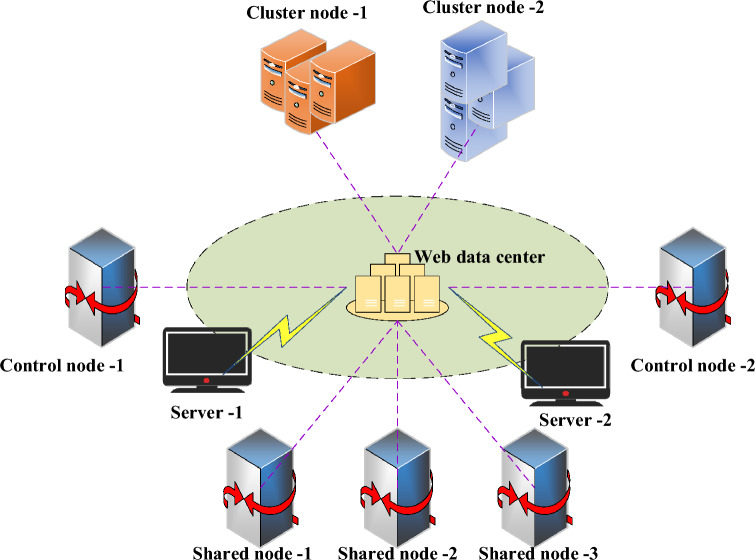
Spatial–temporal distribution structure of blockchain data.

Sensor networks exhibit a dense distribution of effective detection intervals among nodes, leading to spatial correlation and increased spatial redundancy of observed data^28^. The collected data set, however, also display temporal organization in addition to spatial dispersion. Data collected from the same node at different times exhibit variations, resulting in redundancy and resource wastage. When calculating the temporal factor, by considering the spatial correlation as the geographical distribution and the temporal correlation as the temporal variation of the monitoring phenomenon, the spatial and spatio–temporal redundancies can be screened to enhance data collection efficiency. The temporal correlation of the monitoring phenomenon is referred to as the data’s temporal correlation once the spatial coordinates have been determined. To enhance data collection efficiency, it is possible to screen the spatial dimension and spatio–temporal redundancies by compressing the associated data, taking into account the time-related properties of sensor network data. The integration of blockchain technology into database mechanisms can leverage research on time-related characteristics and energy-efficient data collection mechanisms to yield significant benefits. The development of blockchain technology in database mechanisms offers opportunities to explore time-related features and energy-saving data collection mechanisms. Evaluating the suitability of different blockchain approaches involves comparing their performance in terms of time, security, and scalability. Blockchain based on proof of work provides high security but comes with the drawbacks of high energy consumption and slow processing speed. Conversely, blockchain based on proof of stake demonstrates the higher energy efficiency and faster transaction processing, albeit with potential challenges related to decentralization and security. Furthermore, exploring other blockchain consensus algorithms and technologies, such as delegated proof of stake and practical Byzantine fault tolerance, can shed light on their advantages and limitations within a database environment. By conducting a comparative analysis of these blockchain approaches, it becomes possible to select the most suitable solution for specific application scenarios, facilitating more efficient data management and energy utilization. Figure 1 illustrates the organization of blockchain data’s regional and temporal distribution structure.

### Energy-saving mechanism of blockchain and real-time database

**Figure 2:**
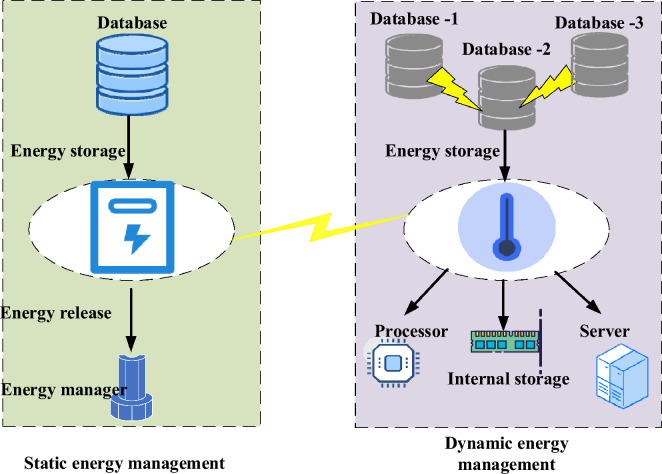
Energy storage structure display of static and dynamic databases.

The blockchain’s data structure consists of two main components: the block title and the block. Within the real-time database system, the block header holds essential metadata, including details about multiple transaction records, block height, current block generation timestamp, a pointer to the main block containing the character string, difficulty level of energy-saving mechanism implementation, and a random number for the current block generation^29,30^. Each transaction record represents a data structure that contains crucial information, such as the sender and recipient details, dates, digital signature value, and hash value. The dynamic collection of network topology data is achieved by utilizing the link-state advertisement protocol mode and the Hello protocol. These protocols enable the system to determine the optimal link state while the router is operational, which involves assessing the activity status of nearby routers and the overhead of data to neighboring nodes through the home packet. Additionally, two prominent research areas in the field encompass the trustworthy service provisioning for three-party anonymous identity authentication in the industrial IoT, supported by blockchain, and the distributed trusted multidomain collaboration of 5G and beyond mobile edge computing, based on blockchain. Establishing a trustworthy service provisioning framework on the blockchain makes it possible to achieve security and reliability in anonymous identity authentication and data interaction. Moreover, the utilization of blockchain in 5G and beyond mobile edge computing facilitates cross-domain collaboration and resource sharing, resulting in improved network efficiency and data processing capabilities. The database energy storage comprises two categorized structural forms: static and dynamic. Figure 2 presents the graphical representation of these structural forms.

### Analysis of database energy-saving mechanism and cluster framework with balanced energy consumption

**Figure 3:**
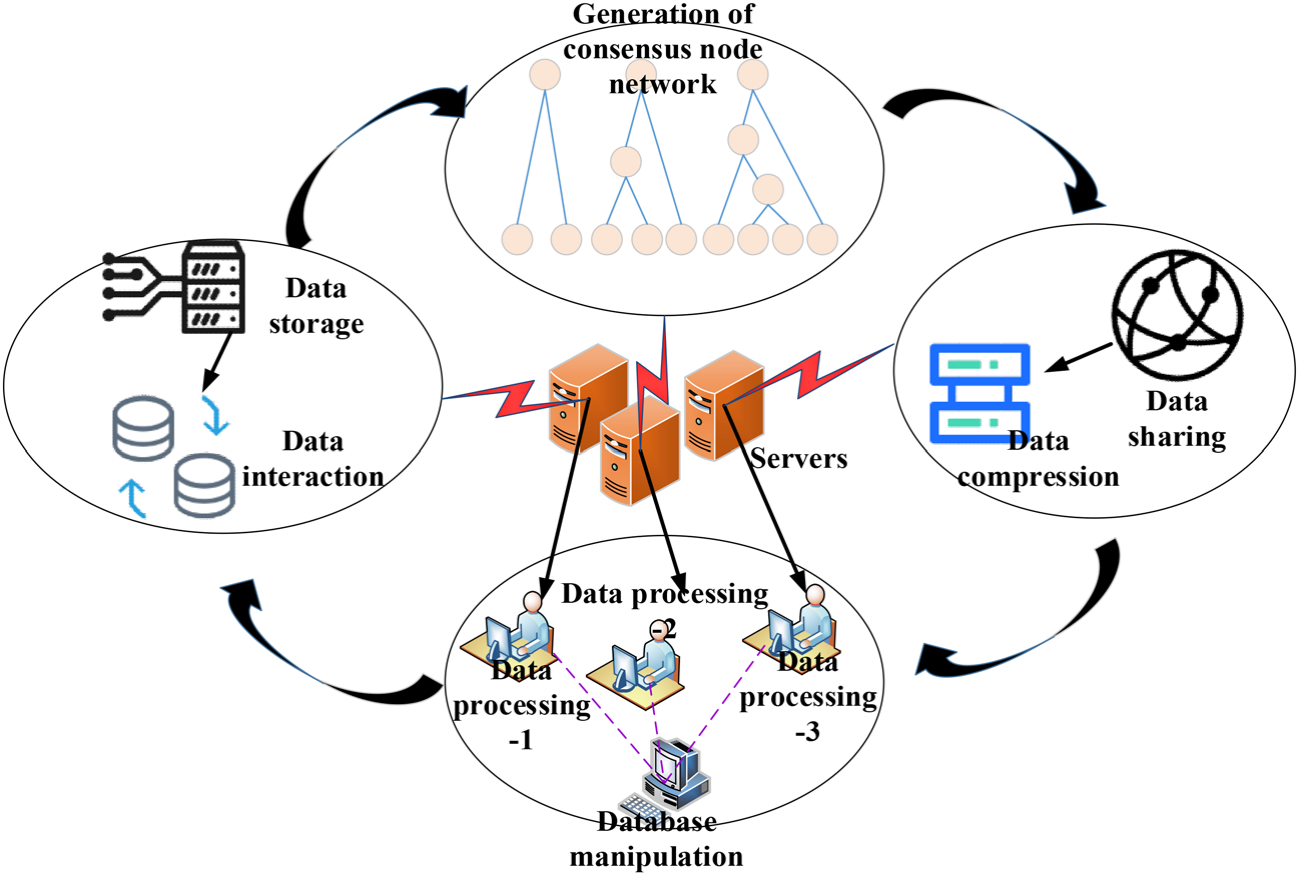
Framework structure of database cluster with load balancing.

The shared and cluster storage architectures are widely used as primary database cluster architectures. However, each database architecture has its drawbacks in terms of energy balancing. Figure 3 illustrates the structure of the load-balancing database cluster framework after analysis.

### Data processing and model analysis

A heterogeneous database cluster prototype system is established to conduct a more realistic experiment. This system comprises a control node, five background nodes, each with varying processing capabilities, and a real-time energy consumption detector to measures the system’s energy usage. Interconnecting all components is a TP-link switch, and the Wake-on-Lan technology allows the control node to reawaken dormant nodes when needed. Table 1 displays the nodes’ hardware setup and energy usage metrics.

**Table 1:** Experimental environment settings.

Experimental environment	Experimental group-1	Experimental group-2	Experimental group-3
Operating system	Windows S10 system with 64-bit	Windows S10 system with 64-bit	Windows S10 system with 64 bits
The central processing unit (CPU)	Intel(R)Core(TM)i7-7700	Intel(R)Core(TM)i5-7700	Intel(R)Core(TM)i3-7700
CPU main frequency	3.70 GHz	3.50 GHz	3.30 GHz
Internal storage	16 GB	16 GB	16 GB
Display card	NVIDIA GeForce GTX 1080 × 4	NVIDIA GeForce GTX 1080 × 4	NVIDIA GeForce GTX 1080 × 4
Storage hard disk	5.2 T	5.2 T	5.2 T
Memory	8 GB	8 GB	8 GB

**Figure 4:**
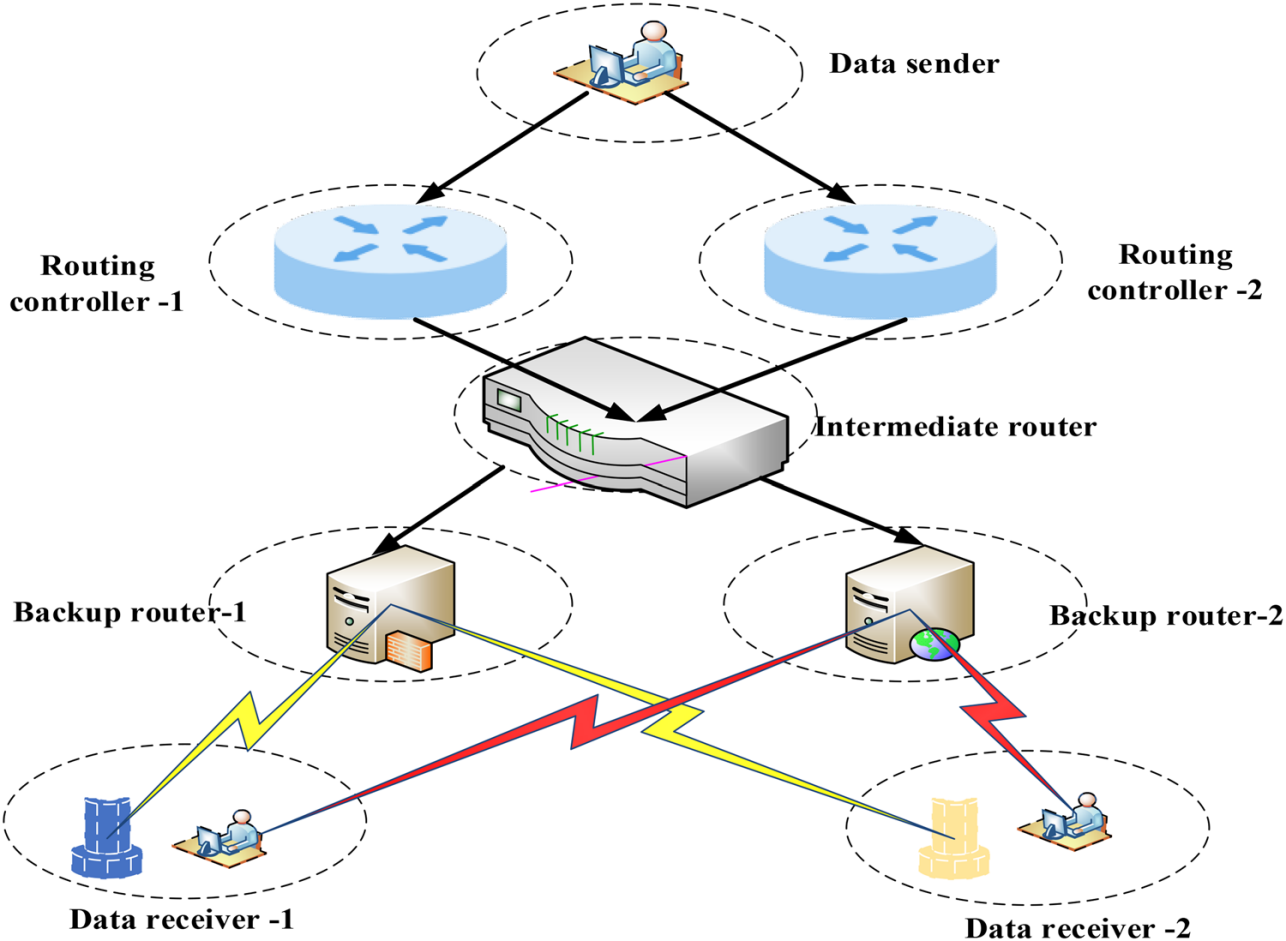
Blockchain data processing and database system transmission process.

In terms of experimental verification, the database energy-saving mechanism within the framework of green environmental protection serves as the most suitable context for the model. The performance indicators to be compared include total system execution time, system query time, average system execution time, database query time, system energy consumption time, and system task processing time. The traditional database adopts a server-based storage approach. Figure 4 illustrates the structure resulting from the analysis of the blockchain data processing and database system transmission process.

In Fig. 4, data is transmitted to various routing controllers via the transmitter and then forwarded to the standby router through the transit router. Finally, different data receivers are used to complete the final reception. The construction strategy of the database energy-saving mechanism is examined based on blockchain technology. Table 2 outlines the technical specifications of the algorithm implementation.

**Table 2:**
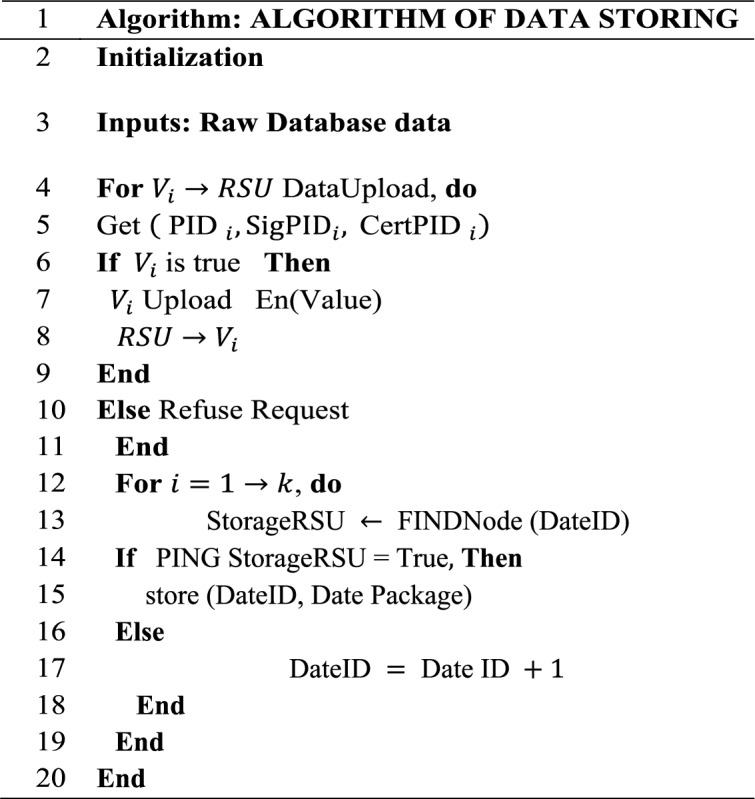
Details of construction technology of database energy-saving mechanism.

## Results and discussion

### Performance analysis of database execution time and query flow

**Figure 5:**
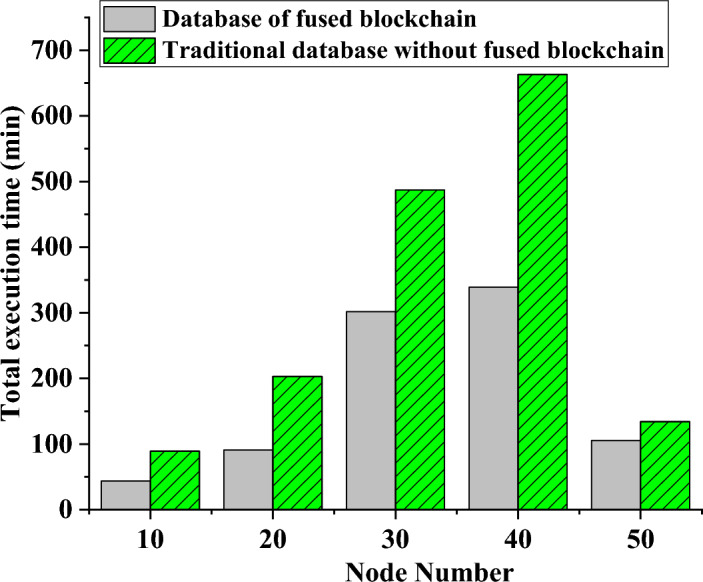
Changing trend of the total execution time of the database system as the number of nodes increases.

**Figure 6:**
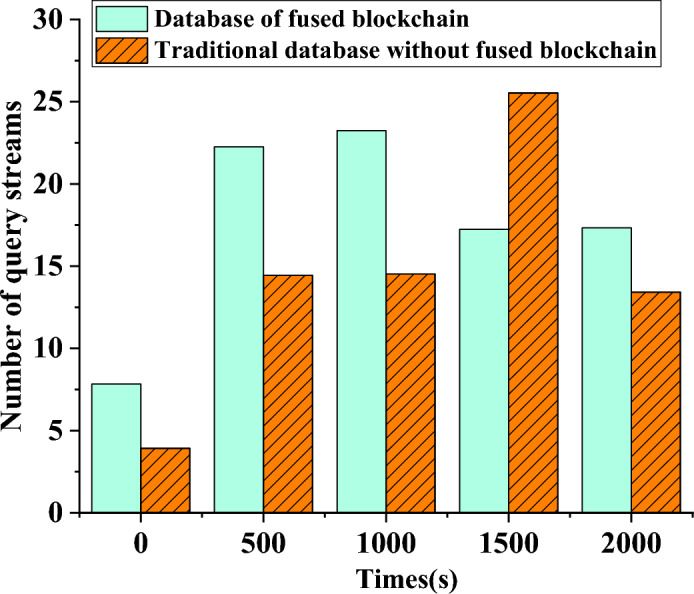
Trends of query flow in different database systems with time.

Through a comparison of the overall command execution time between two database systems with different network nodes, Fig. 5 illustrates the changing trend of the total system execution time. This paper examines the performance disparity between a database system using blockchain technology and a conventional database system in terms of command execution time. Figure 6 displays the variation in query flows of database systems over time, providing a comparison of query flows across different database systems regarding energy savings.

In Fig. 5, the system’s overall execution time fluctuates with the growth of network nodes. Introducing blockchain technology for enhancement can reduce the command execution time to 45 min under the same number of nodes, compared to 98 min under the traditional database method with ten nodes in the system. The combined execution time of two distinct database systems reaches its peak at 40 nodes. Currently, the traditional database system requires 670 min to complete each transaction, whereas the blockchain-based database system can accomplish each transaction in just 340 min.

Figure 6 shows the changing query flow of several database systems over time. At the beginning of the experimental testing period, the standard database mechanism had a query flow of only 3.5 queries per second. In contrast, the database mechanism enhanced with blockchain technology achieved eight queries per second. As the experimental time increased to 1000 s, the traditional database mechanism’s query flow was 15, while the blockchain-optimized database mechanism achieved a query flow of 24. However, as the experimental time continued to increase to 1500 s, the query flow of the traditional database outperformed the blockchain-optimized database. Further analysis of these results may provide valuable insights into the performance and efficiency of different database systems with and without blockchain integration.

In conclusion, the analysis results presented in Figs. 5, 6 align harmoniously with the data articulated in the tables. They unequivocally affirm that, when juxtaposed with conventional databases, blockchain database systems manifest distinct advantages concerning execution efficiency and query throughput. However, it remains imperative to enact further refinements, custom-tailoring these systems to specific application scenarios. The infusion of blockchain technology precipitates a notable reduction in the overall execution time of the database system when equipped with the same number of nodes. It concurrently engenders an augmentation in query throughput within a delimited time frame. Nonetheless, it should be noted that, over protracted durations, the query throughput of traditional database systems may eclipse that of blockchain-optimized counterparts. These findings contribute meaningfully to the comprehensive assessment and comparative analysis of the performance and efficiency inherent in distinct database systems.

### Performance analysis of different types of database network

**Figure 7:**
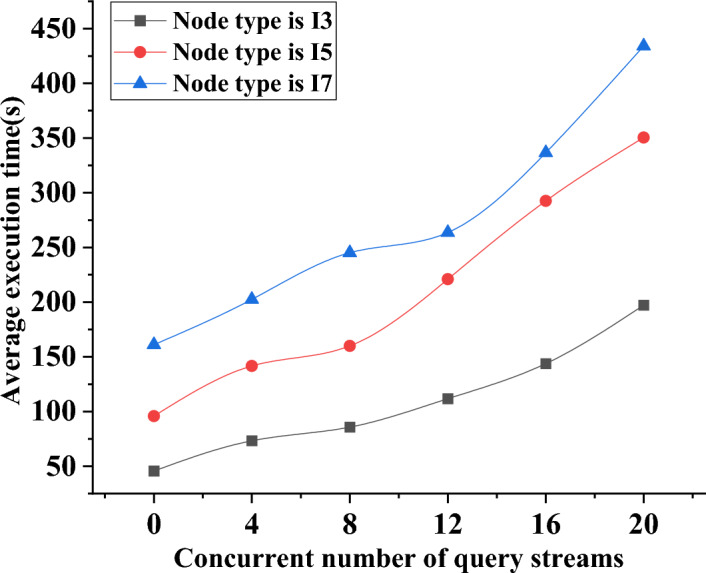
Change trend of the average execution time of different types of networks.

**Figure 8:**
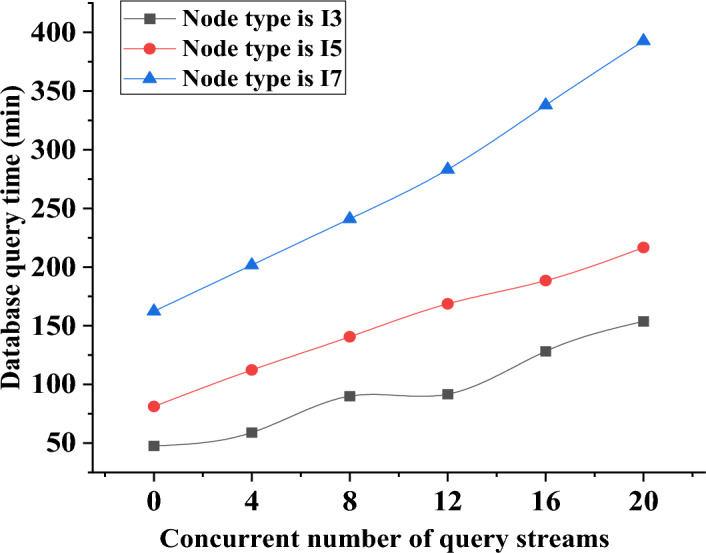
Trends of database query time in different types of networks with the increase of concurrent nodes.

The paper classifies the database network nodes into different categories based on the varying performance of the central processing unit (CPU). The nodes are labeled as I3, representing a CPU frequency of 3.3 GHz, I5 for a CPU frequency of 3.5 GHz, and I7 for a CPU frequency of 3.7 GHz. Figure 7 depicts the variation trend in the execution time of the sorting system by examining the data on the average execution times of different types of database networks. Figure 8 depicts the precise adjustments.

Figure 7 presents a clear relationship between the number of concurrent query streams and the network’s average execution time, considering different CPU frequencies. Initially, with no concurrent query flows, the average execution time is 50 s for an I3 node network, 100 s for an I5 node network, and more than 150 s for an I7 node network. As the network concurrency increases, the average execution time also rises. For instance, with 20 concurrent query flows, the average execution time reaches 200 s for the I3 node network, 350 s for the I5 node network, and over 450 s for the I7 node network. These findings emphasize the importance of adjusting the number of concurrent query flows to optimize the average execution time of the database mechanism. Such adjustments can effectively leverage the energy-saving mechanism and enhance the overall performance of the system.

In Fig. 8, the database query time for various network types demonstrates an increase as the number of concurrent nodes rises. Specifically, the I3 node network exhibits a query time of 50 min, while the I5 node network shows 75 min. In contrast, the I7 node network requires more than 150 min to complete the query when no concurrent nodes are present in the system. Notably, the database query time of the I7 node network experiences the fastest change as the number of concurrent nodes increases. For instance, when there are 16 concurrent nodes in the system, the I7 node database’s query time is 325 min. With further growth to 20 concurrent nodes, the query time for this database’s system data extends to 400 min. Based on careful comparison and analysis, it can be concluded that the database integrated with blockchain exhibits higher system query efficiency compared to the traditional database architecture. These findings highlight the potential of blockchain integration in enhancing the query performance of the database system.

### Performance analysis of execution time and energy consumption of different database systems

**Figure 9:**
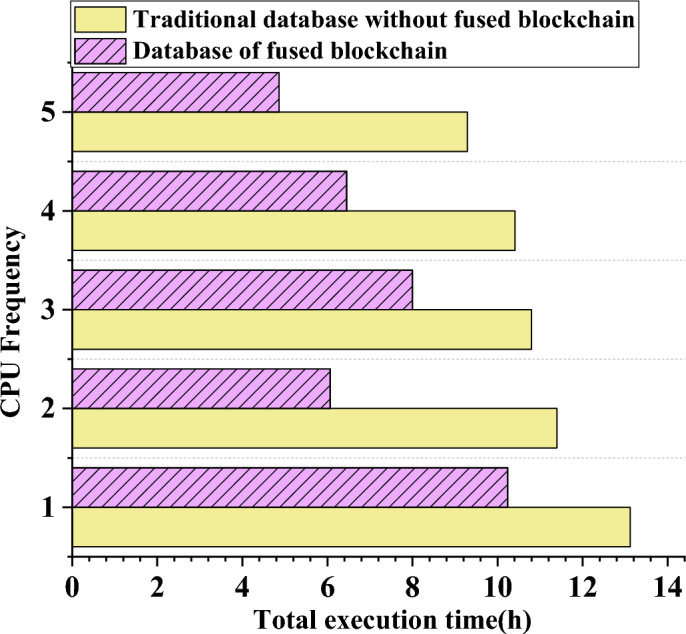
Changing trend of task processing time data of different database systems.

**Figure 10:**
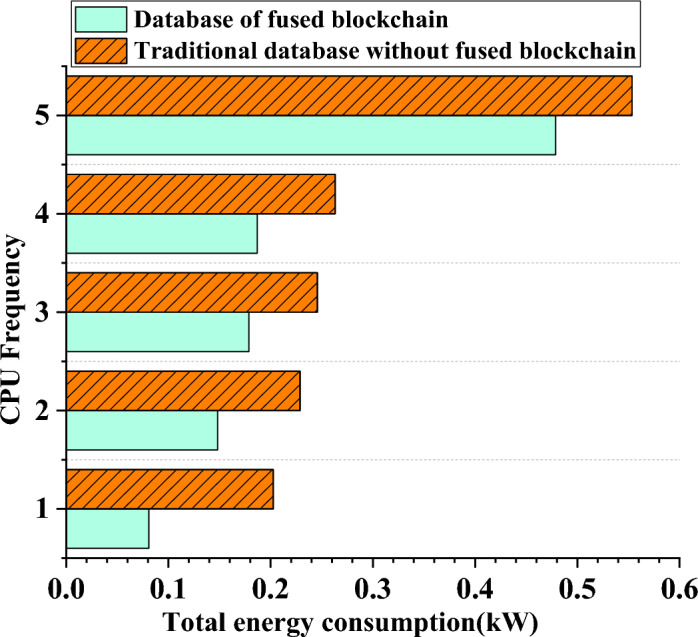
Trends of total energy consumption of different database systems with the increase of CPU frequency.

The data regarding the growth of total execution time and the variations among different database systems have been organized and presented in Fig. 9. In addition, an analysis of the total energy consumption data for the system with increasing host frequencies is displayed in Fig. 10.

In Fig. 9, it is evident that the processing times of numerous database systems significantly decrease as the CPU frequency increases. For example, the task processing time for a conventional database is recorded at 13.2 h when the CPU frequency is set at 1. However, after undergoing data processing and optimization through blockchain technology, the system’s task processing time is reduced to 10 h. Similarly, when the CPU frequency is increased to 3, the job processing time for the traditional database is 10.8 h, while the blockchain-optimized database system accomplishes the same task in just 8 h. Moreover, the database mechanism integrating blockchain technology exhibits the most efficient energy-saving performance when the CPU frequency reaches 5. At this level, the task processing time is 4.8 h, significantly faster than other frequencies, thereby yielding the most substantial energy-saving effect. This efficiency enhancement is attributed to the notably increased computing resource allocation efficiency within the system.

Figure 10 demonstrates that various database systems’ overall energy consumption changes slightly as CPU frequency increases. A traditional database system consumes 0.2 kW of energy when the CPU frequency is 1. In contrast, a fusion database system optimized with the energy-saving mechanism enabled by blockchain technology exhibits a significantly reduced total energy consumption of only 0.08 kW. Although the trend may not be immediately apparent, it becomes evident that the system’s energy usage increases as the CPU frequency rises. Specifically, the regular database uses 0.26 kW of energy when the CPU frequency is 4, while the improved database consumes 0.18 kW. Similarly, at a CPU frequency of 5, traditional databases consume 0.56 kW of energy, whereas optimized databases use 0.48 kW. Consequently, the database system’s ideal CPU frequency is determined to be 4, with the blockchain-powered fused database offering greater energy savings compared to previous database models.

Furthermore, an analysis and comparison of the total execution time and query throughput data for different database systems with varying numbers of nodes were conducted. The results are presented in Tables 3, 4.

**Table 3:** Comparison of total execution time for different database systems.

Database system	10 nodes	20 nodes	30 nodes	40 nodes
Traditional database	98 min	240 min	450 min	670 min
Blockchain database	45 min	150 min	270 min	340 min

**Table 4:** Comparison of query traffic for different database systems.

Database system	1000 s	1500 s	2000s
Traditional database	15 items/second	24 items/second	32 items/second
Blockchain database	8 items/second	18 items/second	22 items/second

Table 3 presents the total execution time for traditional databases and blockchain-optimized databases with varying numbers of nodes. The data reveals that as the number of nodes increases, the execution time for both database types increases. However, it is noteworthy that the blockchain-optimized database system demonstrates a relatively smaller increase in execution time. For example, when there are 10 nodes, the blockchain database system’s execution time is merely 46% of that of the traditional database system. Moreover, as the number of nodes escalates to 40, the blockchain database’s execution time is approximately half that of the traditional database. These findings underscore the efficacy of blockchain technology in enhancing database execution efficiency, particularly in scenarios involving a large number of nodes, where the advantages of blockchain databases are magnified.

Table 4 provides insights into the evolution of query traffic for both database systems over time. Initially, the blockchain database outpaces the traditional database in terms of query traffic. However, as time advances, the query traffic of the traditional database starts to exceed that of the blockchain database. This shift can be attributed to the additional consensus processes inherent in the blockchain database, contributing to a deceleration in query speed. Nevertheless, when considering the entire duration, the blockchain database maintains a certain edge in query traffic compared to the traditional database. Nevertheless, continuous optimization efforts are warranted to enhance query response speed.

### Limitations analysis and discussion

This paper explores the application of blockchain and the IoT in the energy-saving strategy of databases. However, the study also encounters certain limitations. Addressing the scalability of data centers, energy-saving mechanisms, and the transmission performance of distributed databases remains crucial in effectively integrating blockchain technology. While data centers continue to expand, further research is required to explore their seamless integration with blockchain. The established energy-saving methods and cluster query allocation mechanisms in shared and clustered storage architectures lack sufficient experimental data validation to assess their effectiveness. Additionally, optimizing the transmission performance of distributed databases is essential to foster the development of blockchain integration.

In conclusion, this paper presents a preliminary exploration of blockchain and IoT applications in energy-saving strategies for databases but acknowledges its limitations. When compared to previous relevant literature, Asaithambi et al.^31^ proposed an energy-efficient and blockchain-integrated software-defined network (SDN) for industrial IoT, highlighting the advantages in terms of energy efficiency and data privacy. Alkhateeb et al.^32^ conducted an investigation and summary of hybrid blockchain platforms in the IoT domain, addressing scalability, privacy protection, and security issues and proposing research directions and challenges. Kumar et al.^33^ provided a comprehensive survey of blockchain applications in the industrial IoT, emphasizing the need for more detailed descriptions of data center scalability methods and spatial correlation analysis, as well as experimental data to validate energy-saving mechanisms. Further studies are necessary to optimize database transmission performance to accommodate varying query load requirements.

## Conclusion

The continuous growth of information technology and the advent of the big data era have led to an increase in data scale, storage needs, and reaction time demands. Consequently, data centers have expanded in size to accommodate these needs and provide enhanced processing performance. However, this expansion has also intensified the challenge of managing energy consumption more pressing. This paper investigates the integration of blockchain technology with database energy-saving mechanisms to address this issue. The paper focuses on examining the spatial correlation properties of blockchain data and separating the temporal and spatial correlation of stored data based on the current network topology. A cluster query allocation approach and energy-saving mechanism are established within shared and clustered storage architectures. Furthermore, the space–time distribution structure of blockchain data nodes is analyzed. A comparative analysis of different energy storage structures is conducted to evaluate the database energy-saving framework and cluster structure. The experimental results from various groups demonstrate that the system transmission performance of the regional IoT database is superior. Specifically, under the absence of concurrent query flows, the average execution time for an I3 node network is 50 s, 100 s for an I5 node network, and above 150 s for an I7 node network. However, there are several issues with the paper. The primary flaw is that the focus is on computing nodes within the primary cluster architecture. Future studies should explore dynamic modifications of the CPU frequency based on the system load state to enhance the database’s energy-saving effectiveness. Such improvements could potentially lead to even more efficient energy-saving mechanisms.

## Data Availability

All data generated or analysed during this study are included in this published article.
